# Carbapenem-Resistant Bacteria Recovered from Faeces of Dairy Cattle in the High Plains Region of the USA

**DOI:** 10.1371/journal.pone.0147363

**Published:** 2016-01-29

**Authors:** Hattie E. Webb, Marie Bugarel, Henk C. den Bakker, Kendra K. Nightingale, Sophie A. Granier, H. Morgan Scott, Guy H. Loneragan

**Affiliations:** 1 International Center for Food Industry Excellence, Department of Animal and Food Sciences, Texas Tech University, Lubbock, Texas, United States of America; 2 Antimicrobial Resistance Unit, Laboratory for Food Safety, ANSES, Paris-Est University, Maisons-Alfort Cedex, France; 3 Department of Veterinary Pathobiology, College of Veterinary Medicine and Biomedical Sciences, Texas A&M University, College Station, Texas, United States of America; Tianjin University, CHINA

## Abstract

**Objective:**

A study was conducted to recover carbapenem-resistant bacteria from the faeces of dairy cattle and identify the underlying genetic mechanisms associated with reduced phenotypic susceptibility to carbapenems.

**Methods:**

One hundred and fifty-nine faecal samples from dairy cattle were screened for carbapenem-resistant bacteria. Phenotypic screening was conducted on two media containing ertapenem. The isolates from the screening step were characterised via disk diffusion, Modified Hodge, and Carba NP assays. Carbapenem-resistant bacteria and carbapenemase-producing isolates were subjected to Gram staining and biochemical testing to include Gram-negative bacilli. Whole genome sequencing was performed on bacteria that exhibited either a carbapenemase-producing phenotype or were not susceptible to ertapenem and were presumptively Enterobacteriaceae.

**Results:**

Of 323 isolates collected from the screening media, 28 were selected for WGS; 21 of which were based on a carbapenemase-producing phenotype and 7 were presumptively Enterobacteriaceae and not susceptible to ertapenem. Based on analysis of WGS data, isolates included: 3 *Escherichia coli* harbouring *bla*_CMY-2_ and truncated *ompF* genes; 8 *Aeromonas* harbouring *bla*_cphA_-like genes; 1 *Acinetobacter baumannii* harbouring a novel *bla*_OXA_ gene (*bla*_OXA-497_); and 6 *Pseudomonas* with conserved domains of various carbapenemase-producing genes.

**Conclusions:**

Carbapenem resistant bacteria appear to be rare in cattle. Nonetheless, carbapenem-resistant bacteria were detected across various genera and were found to harbour a variety of mechanisms conferring reduced susceptibility. The development and dissemination of carbapenem-resistant bacteria in livestock would have grave implications for therapeutic treatment options in human medicine; thus, continued monitoring of carbapenem susceptibility among enteric bacteria of livestock is warranted.

## Introduction

Carbapenems have become increasingly important as infections caused by pathogenic bacteria resistant to practically all alternative antibiotics have become globally disseminated throughout the past two decades [1, 2]. It is for this reason that carbapenems are reserved as the last resort treatment of multi-drug resistant (MDR) infections in humans. Consequently, the spread of carbapenem-hydrolysing enzymes among Enterobacteriaceae in hospital settings is of grave concern [3–5]. Saliently, an estimated 9,300 healthcare-associated infections due to carbapenem-resistant *Klebsiella* and *E*. *coli* occurr in the United States (US) annually [6]. It is likely that the emergence and dissemination of carbapenem-resistant Enterobacteriaceae (CRE) is the consequence of increased reliance on carbapenems for treatment of MDR infections. Moreover, carbapenem resistance has become an urgent public health concern largely because CRE are commonly co- or cross- resistant to all other clinically relevant antimicrobials; this, in turn, drastically limits—or eliminates—alternative therapeutic options [6, 7].

Carbapenem resistance is generally mediated by two primary mechanisms: (i) production of carbapenemases, enzymes (e.g. KPC, VIM, and OXA) capable of hydrolysing almost all β-lactams or (ii) through modifications to the cell outer membrane, specifically by decreased cell membrane permeability from porin modification, and/or production of efflux pumps; this is often found in combination with cephalosporinase enzymes [7]. Carbapenemases are classified into four different classes (Ambler classes A, B, C, and D) based on their mechanism of antibiotic hydrolysis [8]. The extent of resistance varies among enzymes in that KPC-1 and IMP-1 are commonly associated with resistance to typical therapeutic concentrations of carbapenems, whereas OXA-23 is commonly associated with a reduced susceptibility to carbapenems based on clinical interpretive criteria [9, 10].

In addition to clonal dissemination of CRB, carbapenemase-encoding genes are frequently carried by mobile genetic elements, such as plasmids, and, as a result, carbapenemase encoding genes have been reported across a variety of Enterobacteriaceae genera [11, 12]. Most clinically relevant carbapenem resistance appear to have arisen and propagated as a result of clinical carbapenem use in human medicine [13]; consequently, our knowledge concerning the epidemiology and ecology of carbapenem-resistant bacteria (CRB) is largely limited to human clinical settings.

Carbapenems are not approved for use in livestock production anywhere in the world [14]; as a result, food-animal use is assumed to be rare. Despite—or perhaps because of—the lack of direct selection pressure, little is known about the prevalence of CRB, and more specifically CRE, in livestock populations and their associated environments. Even though there remains a low probability of direct selection, CRB have been reported by investigators in a few instances; for example *bla*_VIM-1_ was detected in *Salmonella* recovered from fattening pig farms and a broiler farm in Germany [15], *bla*_OXA-23_ was detected in *Acinetobacter* isolated from dairy cattle in France and horses in Belgium [16, 17], and *bla*_NDM-1_ was detected in *Acinetobacter* isolates from a chicken farm and from pigs in China [18, 19]. Despite robust existing integrated antimicrobial resistance surveillance systems, surveillance of carbapenem resistance among bacteria of livestock origin is in preliminary stages [20]. Given the high prevalence of co-resistance to other antimicrobial classes among most clinically relevant CREs, it seems highly plausible that, if introduced into livestock populations, CRE would further disseminate to humans through animal contact, the environment, or food. It is important, therefore, to investigate agricultural systems to detect and characterise carbapenem resistance among bacteria of animal origin. The objectives of this project was to recover carbapenem-resistant bacteria (CRB) from the faeces of dairy cattle and identify the underlying genetic mechanisms associated with reduced phenotypic susceptibility to carbapenems.

## Materials and Methods

### Faecal sample collection

Pen-surface faecal samples were collected from a convenience sample of a dairy farm located in southeastern New Mexico, US, and another dairy farm in the Texas Panhandle, US. Permission from the owner of the private operations was received. As pen-floor fecal samples were collection, there was no interaction with vertebrate animals and no approval from an Institutional Animal Care and Use Committee was needed. These dairy farms reported frequent historical use of ceftiofur (a 3^rd^ generation cephalosporin) and we have observed widespread prevalence of genes encoding for AmpC and extended spectrum beta-lactamase (ESBL) enzymes among Enterobacteriaceae (unpublished data). Samples were collected on multiple days from May to July 2014 from pens housing healthy cows in early lactation (maximum 50 days in milk).

### Selective isolation of CRB

Twenty-five grams of faeces were enriched in 225 mL of MacConkey broth (EMD Millipore, Darmstadt, Germany) containing ertapenem (0.5 mg/L; Toronto Research Chemicals, Inc., Toronto, Canada), which was then hand homogenised before incubation at 37°C for 18 to 22 hours. Following incubation, 10 μL was streaked to two agar plates as an initial screening method for CRB, one MacConkey agar (EMD Millipore) plate containing ertapenem (1.0 mg/L; ME) and one modified-SUPERCARB (SC) agar plate. The ME agar was prepared as previously described by Nordmann, Girlich, and Poirel [21]; however, due to the unavailability of Drigalski agar, MacConkey agar instead was used in the SC agar. In addition, the concentration of ertapenem was raised from 0.25 mg/L to 0.5 mg/L, to reflect a change in interpretive criteria for ertapenem [10].

### Phenotypic characterisation of CRB

One colony of each unique morphology was selected and streaked for isolation on tryptic soy agar (EMD Millipore) containing ertapenem (0.5 mg/L; TSAe), which was then incubated for 18 to 22 hours at 37°C (S1 Fig). Isolates from the TSAe were screened in parallel utilising the Modified Hodge test (MHT) and disk diffusion (DD) susceptibility test with an ertapenem-containing disk (10 μg, BD BBL^™^ Sensi-Disc™, Sparks, MD) as previously described [10]. Isolates were further characterised if they exhibited enhanced growth on MHT or else were not susceptible to ertapenem using the Clinical and Laboratory Standards Institute (CLSI) DD interpretive criteria for Enterobacteriaceae, the initial genera targeted [10]. Intermediate and true clinical resistance according to CLSI criteria will herein be described as resistance for simplicity. A Gram stain was performed and presumptive species identification was obtained on all Gram-negative bacilli using biochemical identification test kits specific for Enterobacteriaceae (API 20E strips, Biomérieux, Marcy-l’Etoile, France) and Gram-negative organisms (Sensititre GNID, TREK Diagnostic Systems Inc., Cleveland, OH). To confirm the carbapenemase-positive phenotype, a pH-based test (Carba NP) was performed to confirm hydrolysis of the carbapenem β-lactam as previously described [22]. Following genotypic exploration of genus (described below), the DD results were later reinterpreted using the appropriate criteria where available (as indicated in Table 1) [10]. As criteria have not yet been established for some complexes of *Aeromonas* or for *Pseudomonas* spp. other than *P*. *aeruginosa*, CLSI criteria that are available were used for reinterpretation of the *A*. *allosaccharophila* and *Pseudomonas* spp. isolates reported herein. These values are not meant to imply clinical efficacy, but were interpreted to serve as a phenotypic description in reference to better described relatives.

**Table 1:** Phenotypic and genotypic description of the carbapenemase-producing or ertapenem non-susceptible isolates from dairy cattle faeces that were subjected to WGS.

Isolate	Media^a^	Modifie Hodge Test	Carb NP	Disk Diffusion	Taxonomic Designator (ANIb/16S)	GenBank Accession	Potential Mechanism of resistance
008A	SC	1	1	S*	*Pseudomonas citronellolis*	LKJO00000000	See S1 Table
011A	SC	1	1	S*	*P*. *citronellolis*	LKKN00000000	See S1 Table
066A	SC	0	—	R*	close to *P*. *stutzeri*	LKKJ00000000	See S1 Table
080A	SC	0	—	R*	close to *P*. *anguillispectica*	LKKK00000000	See S1 Table
096B	SC	0	—	R*	close to *P*. *stutzeri*	LKKL00000000	See S1 Table
105A	SC	1	1	S*	close to *P*. *stutzeri*	LKKM00000000	See S1 Table
108A	ME	1	1	I	*Aeromonas veronii*	LKJN00000000	*bla*_*cphA*_
108A	SC	1	1	S	*A*. *veronii*	LKJP00000000	*bla*_*cphA*_
113A	ME	0	—	I	*A*. *veronii*	LKJQ00000000	*bla*_*cphA*_
115A	ME	1	1	I	*A*. *veronii*	LKJR00000000	*bla*_*cphA*_
115A	SC	1	1	I	*A*. *veronii*	LKJS00000000	*bla*_*cphA*_
120B	ME	0	—	R	*Escherichia coli* ST-227^b^, ST-638^c^	LKJT00000000	*bla*_*CMY-2*_ + Tn*ompF + ompC*
121A	ME	0	—	R	*E*. *coli* ST-227^b^, ST-638^c^	LKJU00000000	*bla*_*CMY-2*_ + Tn*ompF + ompC*
125A	SC	1	1	I	*A*. *veronii*	LKJV00000000	*bla*_*cphA*_
130A	ME	1	1	S	*A*. *veronii*	LKJW00000000	*bla*_*cphA*_
130A	SC	1	1	R	*A*. *veronii*	LKJX00000000	*bla*_*cphA*_
131A	ME	1	1	I	*Acinetobacter baumannii* ST-1133^b^, ST-742^c^	LKJZ00000000	*bla*_OXA-497_
131A	SC	1	1	R	*A*. *veronii*	LKJY00000000	*bla*_*cphA*_
134A	ME	1	1	I	*A*. *veronii*	LKKA00000000	*bla*_*cphA*_
134A	SC	1	1	I	*A*. *veronii*	LKKB00000000	*bla*_*cphA*_
140A	SC	1	1	R	*A*. *veronii*	LKKC00000000	*bla*_*cphA*_
141A	ME	1	1	R	*A*. *veronii*	LKKD00000000	*bla*_*cphA*_
141A	SC	1	1	R	*A*. *veronii*	LKKE00000000	*bla*_*cphA*_
142A	SC	1	1	R	*A*. *veronii*	LKKF00000000	*bla*_*cphA*_
143A	ME	1	1	R	*A*. *veronii*	LKKG00000000	*bla*_*cphA*_
143A	SC	1	1	R	*A*. *veronii*	LKKH00000000	*bla*_*cphA*_
148A	ME	0	—	R	*E*. *coli* ST-227^b^, ST-638^c^	LKJM00000000	*bla*_*CMY-2*_ + Tn*ompF + ompC*
159A	SC	1	1	S**	*Aeromonas allosaccharophila*	LKKI00000000	*bla*_*cphA*_

^a^,SC, modified-SUPERCARBA media; ME, MacConkey agar containing ertapenem (1.0 mg/L)

^b^, Oxford scheme;

^c^, Pasteur scheme; 0, carbapenemase-negative; 1, carbapenemase-positive; —, test not performed; S, susceptible; I, intermediate; R, resistant.

*, Interpreted using CLSI carbapenem breakpoints established for *Pseudomonas aeruginosa*;

**, interpreted using CLSI carbapenem breakpoint established for *Aeromonas* spp.; Tn, premature stop codon.

### WGS and bioinformatics analyses

Whole-genome sequencing was performed for: (i) all potential CRE from all screening methods; and (ii) isolates of other families with a carbapenemase-producing phenotype (S1 Fig). Genomic DNA was extracted (Bacterial Genomic Miniprep kit, Sigma-Aldrich, St. Louis, MO), and paired-end libraries were generated (Nextera XT library kit v2, Illumina Inc., San Diego, CA). The resulting libraries were sequenced using the sequencing by synthesis method on an Illumina sequencing platform (MiSeq V.3.0, Illumina Inc., San Diego, CA) to obtain 300 bp reads. A targeted genome coverage of 65x was used given an assumed mean genome size of 5 Mb for 28 isolates per run. Reads were assembled into draft genomes (A5 pipeline [23]) and preliminarily annotated (Prokka [24]). The National Center for Biotechnology Information Prokaryotic Genome Annotation Pipeline (NCBI PGAP [September 23, 2015]) was used for the final genome annotation. Species identification was performed by calculating the average nucleotide identity (ANIb) values between draft genomes obtained in this study and publicly available reference genomes (JSpecies v1.2.1 [25]), and additional phylogenetic confirmation using 16S sequences from draft genomes sequenced at Texas Tech University were compared to type strains from the Ribosomal Database Project (https://rdp.cme.msu.edu/). *Acinetobacter baumannii* and *Escherichia coli* isolates were further typed by multi-locus sequence typing (MLST; MLST 1.8, Center for Genomic Epidemiology [CGE], Lyngby, Denmark). Isolates were screened for antimicrobial resistance genes (SRST2 [26]) using the Antibiotic Resistance Gene-ANNOTation tool (ARG-ANNOT [27]) database and the online tool ResFinder 2.1 (https://cge.cbs.dtu.dk//services/ResFinder/ [28]). Where no putative mechanisms of carbapenem resistance were identified, conserved domain BLASTs were performed using the Integrated Toolkit for Exploration of microbial Pan-genomes (ITEP [29]) in order to identify conserved domains associated with antibiotic resistance genes in other prokaryotes.

Outer membrane protein (OMP) gene sequences, specifically the *ompF* and *ompC*, were extracted from the *E*. *coli* draft genomes sequenced in this study, as well as from a number of reference genomes (Genbank U00096.3, CP008957.1, CP009072.1, NC_013361.1, and HG428755.1). The OMP genes of the carbapenem-resistant *E*. *coli* were compared to the OMP genes of the reference genomes to order to identify possible mutations, truncations, and insertions that might affect protein function.

## Results

From 159 faecal samples, a total of 151 and 172 isolates were collected from the SC and ME plates, respectively. Of these 323 isolates, 163 isolates were deemed of interest as follows: 6 demonstrated a carbapenemase-producing phenotype on the MHT but were ertapenem-susceptible via DD, 142 were ertapenem-resistant via DD, and 15 demonstrated a carbapenemase-producing, ertapenem-resistant phenotype. All DD interpretations at this stage were completed utilising the CLSI Enterobacteriaceae interpretive criteria as a screening method [10]. Within these isolates of interest, 131 were Gram-negative bacilli and, by using the selection criteria (S1 Fig), 28 isolates from 21 pen-surface samples were selected for characterisation by WGS. Twenty-one of the 28 isolates were selected based on a carbapenemase-producing phenotype (regardless of its biochemical characterisation) and 7 presumptive CRE based on DD. Of the isolates selected for WGS, a total of 17 and 11 isolates were collected from SC and ME agar plates, respectively.

As a majority of the biochemical results lacked single genera exclusivity in their results (data not reported), genome interrogation was used for final speciation; the isolates included *Aeromonas* spp. (n = 18), *A*. *baumannii* (n = 1), *Pseudomonas* spp. (n = 6), and *E*. *coli* (n = 3; Table 1). The sequence types (ST) of all *E*. *coli* isolates (n = 3) were identical; that is, either ST-227 (n = 3) or ST-638 (n = 3) depending on the typing scheme used, Oxford or Pasteur [30, 31], respectively. All 3 *E*. *coli* isolates harboured the AmpC β-lactamase gene *bla*_CMY-2_. Comparison of the *ompF* gene sequences in all 3 *E*. *coli* isolates to reference sequences revealed a single base pair insertion at position 373 that resulted in a frameshift and; thus, a predicted premature stop codon in the protein leading to a truncated 128 amino acid OmpF (compared to 362 amino acids of the reference strains). Two of the three *E*. *coli* isolates (120BME and 121AME) showed 100% similarity in their *ompC* amino acid sequences and no predicted premature stop codons were identified. The genomic region containing *ompC* in the remaining *E*. *coli* isolate (148AME) could not be adequately assembled.

A carbapenemase-producing phenotype was observed in the single *A*. *baumannii* and this isolate was of a novel ST designated ST-1133 and ST-742 based on the Oxford [32] and Pasteur [33] scheme, respectively (PubMLST database; http://pubmlst.org, date: October 21, 2015 and September 11, 2015). This isolate harboured an Ambler class D carbapenemase that is part of the OXA-51-like enzyme group (S3 Fig) with 5 nucleotide changes that yield 3 amino acid changes (N111D, D123N, and E227G) from the previously described *bla*_OXA-379_ (KF986260). The observed *bla*_OXA_ β-lactamase gene appeared to be novel and was designated *bla*_OXA-497_ (Lahey β-lactamase database; http://www.lahey.org/Studies/, date: July 8, 2015). It was found in a contig that has high sequence similarity to the sequence of chromosome *A*. *baumannii* CIP70.10 (LN865143.1) and genes flanking *bla*_OXA-497_ did not show similarity to transposable elements (e.g. insertion sequences). The isolate exhibited intermediate resistance to ertapenem using clinical interpretive DD criteria for the *Acinetobacter* spp [10].

Eighteen *Aeromonas* isolates were sequenced; 17 harboured a carbapenemase-producing phenotype that was initially observed on the MHT and was subsequently confirmed with the Carba NP assay. The remaining isolate showed biochemical and phenotypic characteristics of a CRE. Following genome sequencing, 17 isolates were identified as *A*. *veronii* and one isolate as *A*. *allosaccharophila*; the latter isolate exhibited a carbapenemase-producing phenotype, but was susceptible to ertapenem via DD using interpretive criteria for *Aeromonas* spp. of the *A*. *caviae*, *A*. *hydrophila*, and *A*. *veronii* complexes [34]. All of the *A*. *veronii* isolates harboured a *bla*_cphA_-like gene in a highly conserved part of the chromosome, although three allele variations of this gene were identified (S2 Fig).

Of the 6 *Pseudomonas* isolates, 2, 3 and 1 were identified as *P*. *citronellolis*, a putatively undescribed species closely related to *P*. *stutzeri*, and a putatively undescribed species closely related to *P*. *anguillispectica*, respectively. Three of these *Pseudomonas* isolates were considered of interest due to presumptive carbapenemase production (008ASC, 011ASC, and 105ASC), while the remaining three were speculated to be presumptive CRE based on disk diffusion and biochemical characterisation. The initial biochemical findings suggested the possibility that they belonged to the Enterobacteriaceae family; however, these were subsequently identified as *Pseudomonas* spp. based on analysis of sequence data. When utilising the interpretative criteria for *P*. *aeruginosa*, the non-carbapenemase-producing isolates had a reduced susceptibility to ertapenem, whereas those with a carbapenemase-positive phenotype exhibited susceptibility. Identified conserved domains of genes included those encoding Ambler class B (*bla*_IMP_), class C (*bla*_*ampC*_), and class D (*bla*_OXA_) β-lactamases; yet their role in the observed phenotypes remains uncertain (S1 Table).

## Discussion

In this study, we recovered CRB from cattle faeces. Whereas we did not attempt to identify dissemination beyond the dairy farms, our find warrants further consideration of the diversity of CRB on dairies. We selected the 2 dairy farms based on frequent ceftiofur use and previously observed frequency of AmpC and ESBL gene harbourage among Enterobacteriaceae bacteria (unpublished data). We hypothesised, therefore, that the selective pressure of ceftiofur use [35] may have favoured dissemination of these β-lactamases and might also select for carbapenemases, if present. While our report is not intended to be generalised to the wider population of dairy cattle, it does provide novel insights into the diversity of carbapenem-resistance mechanisms and the diversity of bacteria that harbour them in agricultural environments.

Our initial goal was to detect carbapenem resistance among Enterobacteriaceae and we were able to identify 3 CRE isolates of *E*. *coli* species from 159 samples; hence, we conclude that the burden of CRE is extremely low. Moreover, these CRE were negative for carbapenemase production and their expressed phenotypic resistance might be due to a multifactorial mechanism. The combined presence of *bla*_*CMY-2*_ and a lack of OmpF and OmpC have been previously associated with imipenem resistance in *E*. *coli* [36, 37]; however, a more recent report refutes the involvement of OmpC in carbapenem-resistance and suggests that there may be other unknown mechanisms [38]. In the recovered *E*. coli, we were able to confirm the presence of a *bla*_*CMY-2*_ and a premature stop codon in the *ompF;* although the clinical importance of these carbapenemase-negative CRE to human health outcomes remains uncertain.

While not included in our initial goal, we also identified carbapenemase-producing bacteria among non-Enterobacteriaceae families. These were included based on our decision tree (S1 Fig); for some, biochemical test results indicated the possibility that these isolates belonged to the Enterobacteriaceae family. While our biochemical assays might not always have provided conclusive identification, it enabled us to characterise carbapenemase enzymes or carbapenem-resistance among a diverse set of bacteria. In particular, we recovered an *A*. *baumannii* harbouring a novel *bla*_OXA_ gene (*bla*_OXA-497_) that appears to be chromosomally encoded, thus presumably likely to be transferred than if associated with genetic mobile elements, such as plasmids or insertion sequences. Carbapenemase activity by *bla*_OXA_ enzymes can vary as the activity can depend on the extent of gene expression and/or the presence of other resistance mechanisms, such as decreased permeability or efflux [39]. Future investigations to characterise this isolate and to describe the potential mobilisation of *bla*_OXA-497_ and its extent of expression are underway. In addition to *A*. *baumannii*, we recovered many *Aeromonas* isolates harbouring a *bla*_cphA_-type metallo-β-lactamase, which are reported to be common among *A*. *veronii* [40, 41]. The genes encoding cphA-type enzymes are on the chromosome; thus, they might be of less epidemiologically relevance to the dissemination of genes that hydrolyse carbapenems.

In some of the recovered *Pseudomonas* isolates, conserved domains of *bla*_IMP_- and *bla*_OXA_-like genes were identified that could represent an acquired ability to produce carbapenemases of clinical importance. Compared to *P*. *aeruginosa*, little is known about antimicrobial resistance in other species of *Pseudomonas*; thus, it is difficult to make inferences regarding the potential clinical importance of these isolates [10, 42]. Furthermore, there are no clinical interpretive criteria for pseudomonads other than *P*. *aeruginosa*. Interestingly, the presence of conserved domains of carbapenemase-producing phenotype did not always align with the carbapenemase-producing phenotype. While we find proteins with these conserved domains, these proteins appear to have a different function. This could also be due to a discrepancy in the phenotypic result or the presences of unidentified mechanisms conferring carbapenemase production [43, 44].

An important consideration is that universally accepted phenotypic methods to recover and characterise carbapenem resistant (or at least, non-susceptible) bacteria are in early stages of development and, thus, limitations were inherent in the methods used in our study. First, the concentration of ertapenem in the enrichment and selective media may have been too great for isolation of some carbapenemase-producing bacteria; in particular, bacteria producing OXA-like enzymes that have been associated with low-level resistance—due to their weak ability to hydrolyse carbapenems—might not have been recovered [45, 46]. In addition, as our scope of interest broadened *posteriori* to include *Aeromonas* spp., *Acinetobacter* spp., and *Pseudomonas* spp., screening with an alternative carbapenem may have been more appropriate as ertapenem exhibits poor activity on the latter two genera [43, 47]. Finally, the carbapenemase screening method used (the MHT) has been associated with interpretation challenges that include both false-positive and false-negative results [10, 48, 49]. In this study, the Carba NP test was used to confirm carbapenemase production after the MHT; however, it is possible that some carbapenemase-producing bacteria may have been wrongfully disregarded due to interpretation error on the MHT.

Based on the results described herein—and despite the limitations of the study, CRE appear to be very rare among dairy cattle in the High Plains of the USA. Nevertheless, CRB were present in the dairy cattle population sampled. Importantly, many of the carbapenemase genes recognised for their rapid acquisition and dissemination throughout hospital-settings worldwide, e.g. variants of *bla*_KPC_ or *bla*_NDM_ [50], were not found. Despite this, we described a diverse set of bacteria and mechanisms that conferred resistance to carbapenems. These results, therefore, highlight the need for effective approaches to avoid the introduction of clinically relevant genetic elements into cattle populations, and to prevent the emergence of carbapenem resistant determinants in livestock populations through co-selection with other commonly used antimicrobials. Further, we believe there is need for on-going or periodic surveillance of CRB in livestock populations. Clearly, this is contingent on the development of methods with sensitivity and specificity for all types of carbapenemase enzymes. Molecular methods may offer advantages as alternative approaches for the detection of antimicrobial resistance genes; however, given the diversity of genetic mechanisms identified, isolate-based genomic interrogation is still warranted.

## Supporting Information

S1 Fig Decision tree used to determine inclusion criteria for carbapenem-resistant isolates collected from dairy cattle faeces. (PDF)

S2 Fig Phylogeny demonstrating variation of *cphA* gene in *Aeromonas* isolates found in this study. Sequences of *cphA* were aligned using Muscle version 3.8.31 (Edgar, RC. 2004. Nucleic Acids Research, 32(5), 1792–1797) and a parsimony based phylogeny was inferred using PAUP*version 4.0b10 (Wilgenbusch, J. C., & Swofford, D. (2003). Current Protocols in Bioinformatics / Editoral Board, Andreas D Baxevanis [Et Al], Chapter 6, Unit 6.4. http://doi.org/10.1002/0471250953 bi0604s00), using the heuristic search option. Values on the branches are the inferred number of steps (single nucleotide substitutions). The search resulted in 1 tree (317 steps, C.I. = 0.647). Values below the branches indicate bootstrap values based on 500 replicates, values below < 50 are not shown.
(PDF)

S3 Fig Maximum likelihood phylogenies of representative OXA- types and OXA-51-like nucleotide sequences (inset) including the novel OXA-497 (marked in red). Nucleotide sequences were aligned using an amino acid sequence guided strategy using TranslatorX (Abascal et al. 2010. Nucleic Acids Research, 38 Web Server issue), W7–13. http://doi.org/10.1093/nar/gkq291), phylogenies were inferred using RAxML version 8.1.20 (Stamatakis, A. 2014. Bioinformatics 30(9), 1312–1313. http://doi.org/10.1093/bioinformatics/btu033). Values on the branches represent bootstrap values as inferred using the autoFC bootstopping option (Pattengale et al. 2010, Journal of Computational Biology: a Journal of Computational Molecular Cell Biology, 17(3), 337–354. http://doi.org/10.1089/cmb.2009.0179).
(PDF)

S1 Table Overview of putative beta-lactamase like genes in *Pseudomonas* spp. found by conserved domain searches. (DOCX)
