# Supplementary material for: Carbapenem-Resistant Bacteria Recovered from Faeces of Dairy Cattle in the High Plains Region of the USA
Source: PLoS One. 2016 Jan 29;11(1):e0147363. doi: 10.1371/journal.pone.0147363 (PMC4732617; doi:10.1371/journal.pone.0147363)
Supplement: S1 Fig — (PDF) [file pone.0147363.s001.pdf]

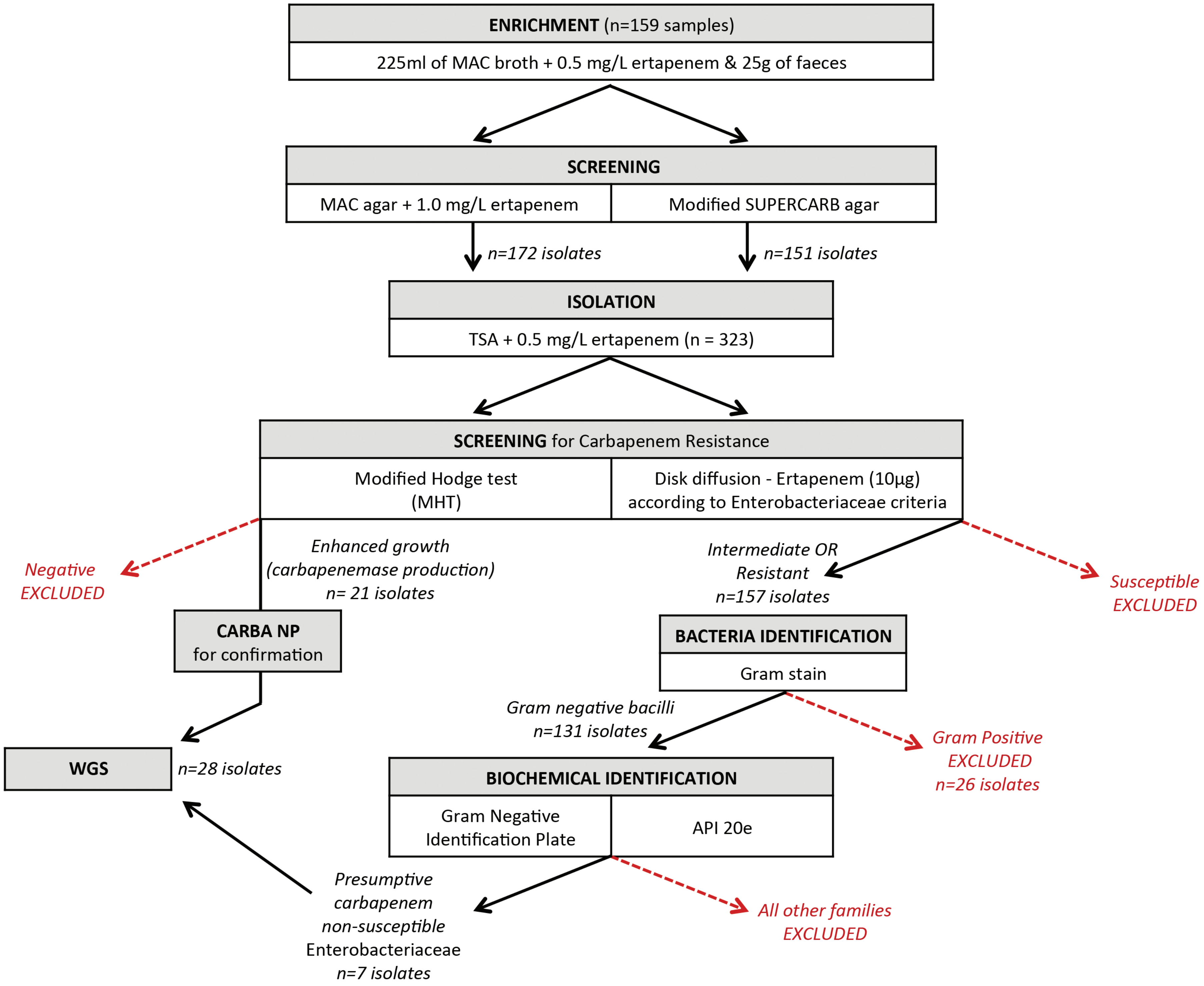

S1 Fig. Decision tree used to determine inclusion criteria for carbapenem-resistant isolates collected from dairy cattle faeces.
