# Supplementary material for: Carbapenem-Resistant Bacteria Recovered from Faeces of Dairy Cattle in the High Plains Region of the USA
Source: PLoS One. 2016 Jan 29;11(1):e0147363. doi: 10.1371/journal.pone.0147363 (PMC4732617; doi:10.1371/journal.pone.0147363)
Supplement: S2 Fig — Sequences of cphA were aligned using Muscle version 3.8.31 (Edgar, RC. 2004. Nucleic Acids Research, 32(5), 1792–1797) and a parsimony based phylogeny was inferred using PAUP*version 4.0b10 (Wilgenbusch, J. C., & Swofford, D. (2003). Current Protocols in Bioinformatics / Editoral Board, Andreas D Baxevanis [Et Al], Chapter 6, Unit 6.4. http://doi.org/10.1002/0471250953 bi0604s00), using the heuristic search option. Values on the branches are the inferred number of steps (single nucleotide substitutions). The search resulted in 1 tree (317 steps, C.I. = 0.647). Values below the branches indicate bootstrap values based on 500 replicates, values below < 50 are not shown. (PDF) [file pone.0147363.s002.pdf]

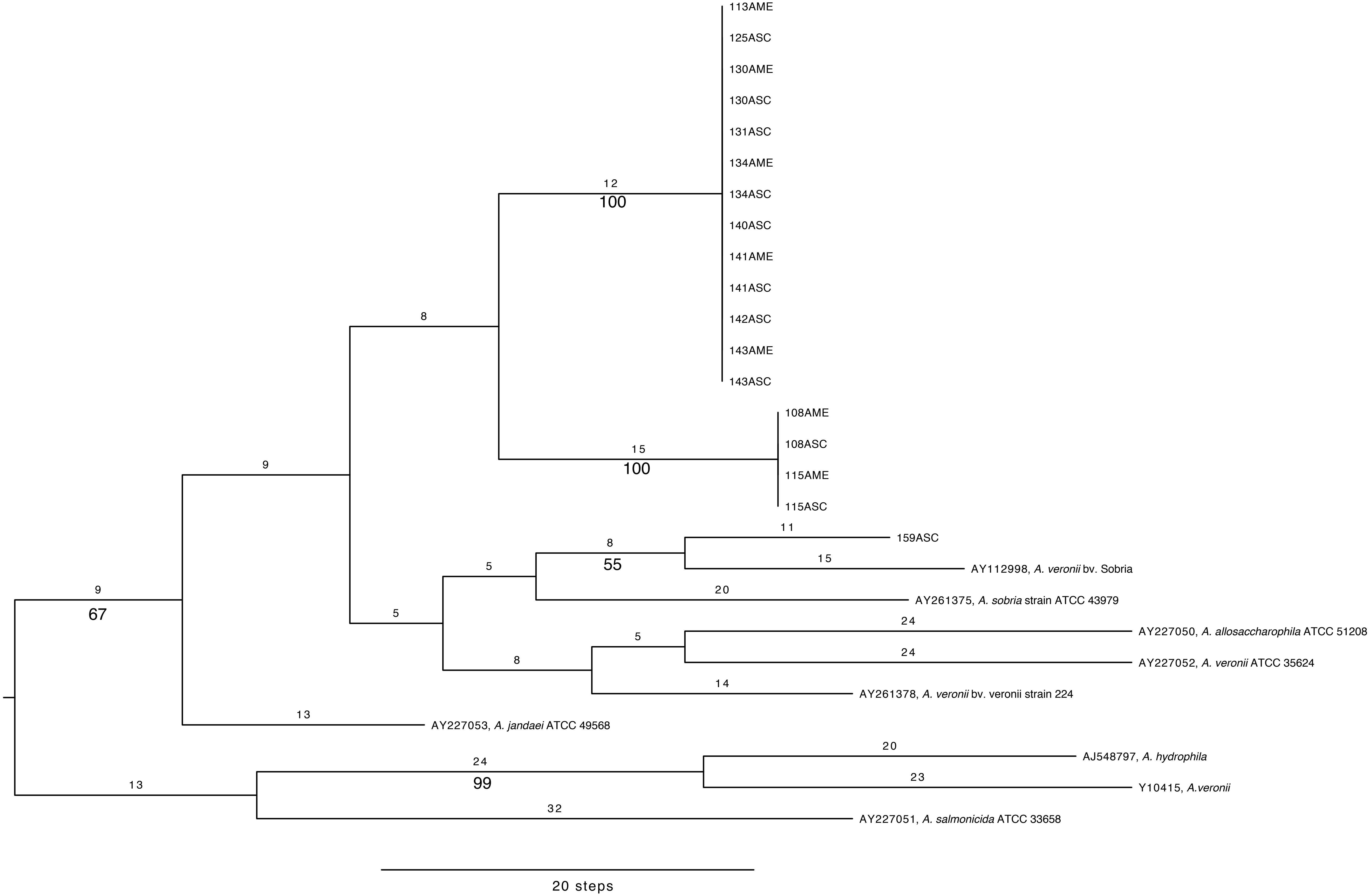

S2 Fig. Phylogeny demonstrating variation of cphA gene in *Aeromonas* isolates found in this study. Nucleotide sequences of cphA were aligned using Muscle version 3.8.31 (Edgar, RC. 2004. Nucleic Acids Research, 32(5), 1792–1797) and a parsimony based phylogeny was inferred using PAUP*version 4.0b10 (Wilgenbusch, J. C., & Swofford, D. (2003). Current Protocols in Bioinformatics / Editoral Board, Andreas D Baxevanis [Et Al], Chapter 6, Unit 6.4. http://doi.org/10.1002/0471250953.bi0604s00), using the heuristic search option. Values on the branches are the inferred number of steps (single nucleotide substitutions). The search resulted in 1 tree (317 steps, C.I.=0.647). Values below the branches indicate bootstrap values based on 500 replicates, bootstrap values below < 50 are not shown.
