# Supplementary material for: Carbapenem-Resistant Bacteria Recovered from Faeces of Dairy Cattle in the High Plains Region of the USA
Source: PLoS One. 2016 Jan 29;11(1):e0147363. doi: 10.1371/journal.pone.0147363 (PMC4732617; doi:10.1371/journal.pone.0147363)
Supplement: S3 Fig — Nucleotide sequences were aligned using an amino acid sequence guided strategy using TranslatorX (Abascal et al. 2010. Nucleic Acids Research, 38 Web Server issue), W7–13. http://doi.org/10.1093/nar/gkq291), phylogenies were inferred using RAxML version 8.1.20 (Stamatakis, A. 2014. Bioinformatics 30(9), 1312–1313. http://doi.org/10.1093/bioinformatics/btu033). Values on the branches represent bootstrap values as inferred using the autoFC bootstopping option (Pattengale et al. 2010, Journal of Computational Biology: a Journal of Computational Molecular Cell Biology, 17(3), 337–354. http://doi.org/10.1089/cmb.2009.0179). (PDF) [file pone.0147363.s003.pdf]

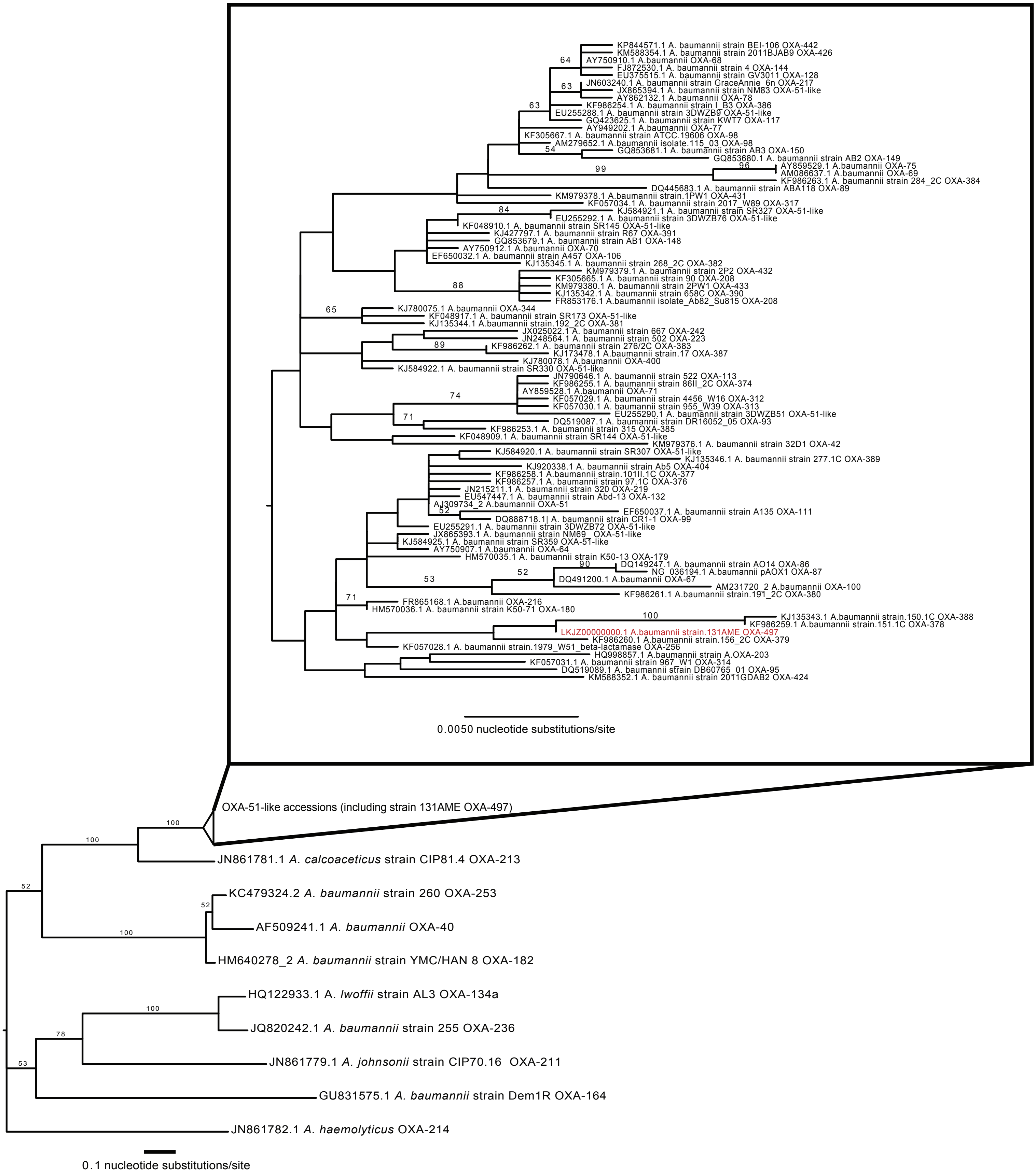

S3 Fig. Maximum likelihood phylogenies of representative OXA- types and OXA-51-like nucleotide sequences (inset) including the novel OXA-497 (marked in red). Nucleotide sequences were aligned using a amino acid sequence guided strategy using TranslatorX (Abascal et al. 2010. Nucleic Acids Research, 38 Web Server issue), W7–13. http://doi.org/10.1093/nar/gkq291), phylogenies were inferred using RAxML version 8.1.20 (Stamatakis, A. 2014. Bioinformatics 30(9), 1312–1313. http://-doi.org/10.1093/bioinformatics/btu033). Values on the branches represent bootstrap values as inferred using the autoFC bootstopping option (Pattengale et al. 2010, Journal of Computational Biology : a Journal of Computational Molecular Cell Biology, 17(3), 337–354. http://doi.org/10.1089/cmb.2009.0179).
