# Supplementary material for: Carbapenem-Resistant Bacteria Recovered from Faeces of Dairy Cattle in the High Plains Region of the USA
Source: PLoS One. 2016 Jan 29;11(1):e0147363. doi: 10.1371/journal.pone.0147363 (PMC4732617; doi:10.1371/journal.pone.0147363)
Supplement: S1 Table — (DOCX) [file pone.0147363.s004.docx]

**S1 Table.** Overview of putative beta-lactamase like genes in *Pseudomonas spp*. found by conserved domain searches.

| Isolate | Putative species^a^ | locus_tag^b^ | Annotation (Prokka) |
| --- | --- | --- | --- |
| 66ASC | close to *P. stutzeri* | PROKKA_02049 | Beta-lactamase precursor ampC |
| 66ASC | close to *P. stutzeri* | PROKKA_02600 | Beta-lactamase IMP-1 precursor |
| 66ASC | close to *P. stutzeri* | PROKKA_02768 | Beta-lactamase Toho-1 precursor bla |
| 66ASC | close to *P. stutzeri* | PROKKA_03686 | Metallo-beta-lactamase superfamily protein |
| 66ASC | close to *P. stutzeri* | PROKKA_03949 | Beta-lactamase HcpA precursor HcpA2 |
| 80ASC | close to *P. anguillispectica* | PROKKA_00143 | Beta-lactamase precursor ampC |
| 80ASC | close to *P. anguillispectica* | PROKKA_00194 | beta-lactamase HcpC precursor |
| 80ASC | close to *P. anguillispectica* | PROKKA_00908 | Metallo-beta-lactamase superfamily protein |
| 80ASC | close to *P. anguillispectica* | PROKKA_01023 | Beta-lactamase HcpA precursor hcpA_1 |
| 80ASC | close to *P. anguillispectica* | PROKKA_01105 | Beta-lactamase 2 precursor |
| 80ASC | close to *P. anguillispectica* | PROKKA_01698 | Beta-lactamase OXA-10 precursor bla |
| 80ASC | close to *P. anguillispectica* | PROKKA_02958 | Metallo-beta-lactamase superfamily protein |
| 96BSC | close to *P. stutzeri* | PROKKA_01407 | Metallo-beta-lactamase superfamily protein |
| 96BSC | close to *P. stutzeri* | PROKKA_01986 | Beta-lactamase precursor ampC |
| 96BSC | close to *P. stutzeri* | PROKKA_03103 | Beta-lactamase IMP-1 precursor |
| 96BSC | close to *P. stutzeri* | PROKKA_03470 | Beta-lactamase Toho-1 precursor bla |
| 105ASC | close to *P. stutzeri* | PROKKA_00452 | Beta-lactamase |
| 105ASC | close to *P. stutzeri* | PROKKA_01542 | Beta-lactamase hydrolase-like protein blh |
| 105ASC | close to *P. stutzeri* | PROKKA_01776 | Beta-lactamase OXA-2 precursor bla |
| 105ASC | close to *P. stutzeri* | PROKKA_03187 | Beta-lactamase precursor ampC |
| 11ASC | *P. citronellolis* | 11ASC_00237 | Metallo-beta-lactamase superfamily protein |
| 11ASC | *P. citronellolis* | 11ASC_01473 | Putative beta-lactamase HcpC precursor, hcpC_1 |
| 11ASC | *P. citronellolis* | 11ASC_01669 | Putative beta-lactamase HcpC precursor, hcpC_2 |
| 11ASC | *P. citronellolis* | 11ASC_03024 | Metallo-beta-lactamase superfamily protein |
| 11ASC | *P. citronellolis* | 11ASC_03088 | Beta-lactamase precursor blaP |
| 11ASC | *P. citronellolis* | 11ASC_03485 | Metallo-beta-lactamase superfamily protein |
| 11ASC | *P. citronellolis* | 11ASC_04169 | Beta-lactamase hydrolase-like protein blh |
| 8ASC | *P. citronellolis* | 8ASC_01448 | Putative beta-lactamase HcpC precursor hcpC_1 |
| 8ASC | *P. citronellolis* | 8ASC_01508 | Putative beta-lactamase HcpC precursor hcpC_2 |
| 8ASC | *P. citronellolis* | 8ASC_01639 | Metallo-beta-lactamase superfamily protein |
| 8ASC | *P. citronellolis* | 8ASC_01726 | Metallo-beta-lactamase superfamily protein |
| 8ASC | *P. citronellolis* | 8ASC_02347 | Beta-lactamase hydrolase-like protein, blh |
| 8ASC | *P. citronellolis* | 8ASC_02980 | Metallo-beta-lactamase superfamily protein |
| 8ASC | *P. citronellolis* | 8ASC_03687 | Beta-lactamase precursor blaP |

^a^ Species identification based on whole genome and 16S sequence data. The designation ‘close to’ is used when a taxon was phylogenetically close the indicated species, but sequence data suggest this may represent a new species.

^b^ Locus tags as obtained from Prokka annotations, and used in the initial analyses. This should be replaced with locus tags designated by NCBI, once we have the annotations back.
